# Stimulus induced resonance in a neural mass model driven with a temporally correlated noise

**DOI:** 10.1186/1471-2202-16-S1-P75

**Published:** 2015-12-18

**Authors:** Maciej Jedynak, Antonio J Pons, Jordi Garcia-Ojalvo

**Affiliations:** 1 Departament de Fısica i Enginyeria Nuclear, Universitat Politècnica de Catalunya, Barcelona, Spain; 2 Department of Experimental and Health Sciences, Universitat Pompeu Fabra, Parc de Recerca Biomèdica de Barcelona, Barcelona, Spain

## 

Power spectra of experimental recordings such as EEG and LFP exhibit a broadband nature characterized by a stable 1/f^b ^background with embedded peaks. These peaks are associated with specific brain states and stimuli, and depending on conditions appear at different frequencies. Interactions between brain rhythms from different frequency bands are assumed to play an important role in brain functioning. Here we study theoretically a particular cross-frequency interaction that has been observed experimentally [1]. That experiment examined the effect of slow rocking on sleep in human subjects, and showed that slow oscillatory stimulus related to rocking enhanced power in EEG measurements in low (0.6-4 Hz) and alpha frequency bands, thus revealing a cross-frequency transfer of power.

We studied this effect by means of a neural mass description based on a model developed by Jansen and Rit [2]. This model typically yields a power spectrum with a narrow peak that corresponds to its intrinsic frequency. In our case, however, a realistic 1/f^b ^spectrum with an embedded alpha peak is observed. This results from a combination of two factors. The first factor is a coexistence (or proximity) of different dynamical regimes near the working point of the model. The second factor is the presence of temporally correlated noise acting upon the model, which enforces flips between different dynamical regimes [3]. Here, we examine systematically the effect of this stochastic driving on the single compartment and on collective effects occurring in the network of interconnected compartments.

We show that the correlation time of the noise is one of the crucial factors that modulate the preference of dynamical regimes for a single compartment, and thus determine the model's activity. Furthermore, we show that this dependence has non-trivial, non-monotonous characteristics. We find that experimental results obtained in the absence of a stimulus are best reproduced when the model operates close to a bifurcation. In the presence of the stimulus, computational results recapitulate the experimental observations when the alpha resonance is not fully developed. The emergence of resonance depends on the driving signal amplitude, properties of the noise, and it relies on collective synchronization, which in turn depends on the coupling between the network elements. The model allows us to make predictions for driving with frequencies greater than the one used in the experiment. Our results indicate that the cross-frequency transfer observed experimentally may occur only for slow driving: for faster input the alpha peak does not increase and might even be suppressed.

In summary, our results show that a 1/f^b ^realistic power spectrum with an embedded alpha peak can be obtained from a neural mass model driven by a temporally correlated noise. The model's dynamics depend in a non-monotonous way on the correlation time of the noise. Slow oscillatory driving of this model operating close to a bifurcation facilitates development of a resonance in the alpha band, giving rise to a cross-frequency power transfer, which reproduces experimental observations [1].
